# Femoral Stem Subsidence in Total Hip Arthroplasty: Comparison between Fixation Principles

**DOI:** 10.1055/s-0046-1825542

**Published:** 2026-10-06

**Authors:** André N. B. M. de Macedo, Lourenço P. Peixoto, João A. M. Guimarães, Conrado T. Laett

**Affiliations:** 1 Divisão de Ensino e Pesquisa, Instituto Nacional de Traumatologia e Ortopedia, Rio de Janeiro, RJ, Brazil; 2 Programa de Pós-graduação em Ciências da Reabilitação, Centro Universitário Augusto Motta, Rio de Janeiro, RJ, Brazil

**Keywords:** arthroplasty, replacement, hip, femur, hip prosthesis, orthopedic, prosthesis design, artroplastia do quadril, desenho de prótese, fêmur, ortopedia, prótese de quadril

## Abstract

**Objective:**

To compare the magnitude of subsidence between single- (SW) and double-wedge (DW) femoral stems in primary total hip arthroplasties (THAs), and to identify factors associated with clinically relevant subsidence.

**Methods:**

Retrospective cohort study with 82 patients (164 hips) undergoing bilateral THA between 2012 and 2022, each receiving type-I (Corail) and -II (Summit) stems, both manufactured by DePuy Synthes. Subsidence was measured radiographically up to 6 months postoperatively and was considered clinically relevant when greater than 3 mm. Radiographic variables included cortical enlargement index (CEI) and canal fill rate (CFR). Statistical analyses included paired t-test, logistic regression, and Spearman correlation.

**Results:**

The mean subsidence was 1.93 ± 1.16 mm in type-I stems and 2.23 ± 1.57 mm in type-II ( *p* = 0.181). Clinically relevant subsidence occurred in 18.2% of type-I stems and 31.7% of type-II (OR = 2.07; *p* = 0.050). Greater subsidence was observed in the first surgery in bilateral arthroplasties ( *p* = 0.006). There was no significant correlation between subsidence, age, body mass index (BMI), CEI, or CFR.

**Conclusion:**

The DW stems were associated with a higher risk of clinically relevant subsidence. In bilateral THA, subsidence is more pronounced at first surgery, with no detectable influence of demographic or radiographic factors.

## Resumo

**Objetivo:**

Comparar a magnitude do afundamento entre hastes femorais de cunha única (CU) e dupla (CD) em artroplastias totais do quadril (ATQ) primárias e identificar fatores associados a afundamentos clinicamente relevantes.

**Métodos:**

Estudo de coorte retrospectiva com 82 pacientes (164 quadris) submetidos a ATQ bilateral entre 2012 e 2022, cada um recebendo haste tipo I (Corail) e II (Summit). O afundamento foi mensurado radiograficamente até 6 meses de pós-operatório, sendo considerado clinicamente relevante quando superior a 3 mm. Variáveis radiográficas incluíram o índice de alargamento cortical (IAC) e a taxa de preenchimento do canal (TPC). As análises estatísticas incluíram teste t pareado, regressão logística e correlação de Spearman.

**Resultados:**

O afundamento médio foi de 1,93 ± 1,16 mm nas hastes tipo I e 2,23 ± 1,57 mm nas tipo II ( *p* = 0,181). Afundamentos clinicamente relevantes ocorreram em 18,2% das hastes tipo I e 31,7% das tipo II (OR = 2,07; *p* = 0,050). Observou-se maior afundamento na primeira cirurgia em artroplastias bilaterais ( *p* = 0,006). Não houve correlação significativa entre afundamento e idade, índice de massa corporal (IMC), IAC ou TPC.

**Conclusão:**

As hastes de cunha dupla apresentaram maior risco de afundamento clinicamente relevante. Em ATQs bilaterais, o afundamento é mais pronunciado na primeira cirurgia, sem influência detectável de fatores demográficos ou radiográficos.

## Introduction

Total hip arthroplasty (THA) is considered one of the most successful surgical interventions in modern medicine. The first THAs were from the 1930s, with marked advances in the 1960s thanks to innovations by John Charnley, 1 who introduced the concept of low articular friction by employing small-diameter femoral heads articulated with high-density polyethylene sockets, fixed to the femur with cemented metal stems. Since then, the study of the prostheses' biomechanical properties has driven technical improvements, including modern liners in the acetabular component, porous surfaces that promote cementless fixation, and improved femoral stem design. 1 2 3

The success of THA is influenced by immutable factors, such as femoral morphology and patient habits, 4 in addition to crucial surgical aspects, such as the initial stability of the femoral stem. Stem instability can result in subsidence, which causes dysmetria between limbs, increases the risk of aseptic loosening, and may culminate in pain and the need for revision. 5 6 7 The subsidence is observed mainly in the first 6 months of the postoperative period, 8 and radiographic follow-up is essential. Previous investigations have provided evidence that the collared design, 5 9 adequate lateral cortical contact, 10 and controlled progression of weight discharge 11 contribute to reducing subsidence greater than 3 mm, considered clinically relevant. New investigations are needed to identify additional modifiable factors influencing subsidence, particularly among the Brazilian population, to inform more effective surgical practice.

The stems used in cementless THA techniques are primarily divided into two groups based on different fixation principles. Single-wedge ones (SW, type I), flat in the anteroposterior plane and conical in the medial-lateral one, promote fixation at three points and reduce contact with the femoral diaphysis, minimizing bone resorption by cortical overload. Double-wedge stems (DW, type II) seek greater metaphyseal filling, increasing the support area. 2 3 This increase in the support area, however, can, in turn, increase friction and stimulate bone remodeling, potentially increasing subsidence. 12

In the present study, the Corail (DePuy Synthes) stem was classified as SW (type I), according to Khanuja et al., 3 based on its predominant biomechanical principle of metaphyseal fixation and load transfer, although it presents enlarged proximal geometry in the sagittal and coronal planes, and a quadrangular cross-section.

Given the impact on clinical outcomes, it is essential to evaluate subsidence in femoral stems across different fixation principles. Thus, the present study aims to compare the magnitude of subsidence between SW and DW femoral stems in primary THA and, as a secondary objective, to analyze risk factors for clinically relevant subsidence.

## Methods

The present is a retrospective cohort study with 6 months postoperative follow-up to assess femoral stem subsidence. Patients undergoing THA at the institution between March 2012 and March 2022 were considered, and those with bilateral THA who received type I stem (Corail) in one hip and type II stem (Summit – DePuy Synthes) in the other were included. Inclusion criteria were age between 18 and 75 years at the time of both surgeries and not having undergone other lower-limb surgeries. Patients with loss of radiographic follow-up were excluded. The project was previously approved by the institutional review board, under the CAAE: 68093223.2.0000.5273.

All surgical procedures were performed in a standardized manner. Spinal anesthesia associated with peripheral femoral nerve block was used, guided by electrostimulation. Antibiotic prophylaxis was initiated intravenously with cefazolin, administered 30 minutes before surgical incision. Surgeries were performed by posterolateral approach. Postoperatively, all patients underwent the institutional prophylaxis protocol, with cefazolin for 24 hours and low-molecular-weight heparin for 30 days. for venous thromboembolism prophylaxis.

Radiographic images of the pelvis in the anteroposterior plane obtained at the last preoperative visit, in the immediate postoperative period, and at the outpatient visits of 1, 3, and 6 months postoperatively were analyzed. The subsidence in the follow-up period was measured as the change in femoral stem height relative to the value measured on the immediate postoperative image. In each image, the prosthesis height was obtained by the distance between the shoulder of the femoral stem and the greater trochanter. Subsidence greater than 3 mm at the end of 6 months was considered clinically relevant, as defined in other studies on the subject. 10

Bone quality was evaluated in the preoperative image by the cortical enlargement index (CEI), the ratio between the metaphyseal diameter 2 cm above the lesser trochanter and the diameter of the isthmus. 13 The femoral component's fitting was evaluated in the image of the immediate postoperative period, using the canal fill rate (CFR) at the transition from the proximal to the distal part of the stem (A), 2 cm from the tip of the stem (C), and at the midpoint between the anterior points (B). 13 All radiographic measurements were made using the Vivace Connect 2022 software. Dates of surgery, sex, and Body Mass Index (BMI) were obtained from the medical record.

According to the Shapiro-Wilk test, subsidence and age were normally distributed, being presented as mean ± standard deviation (SD), whereas the other variables were presented as median (interquartile range [IQR]). Subsidence comparison at the end of 6 months between stem types and orders of surgery was made using repeated-measures t-tests. The effect on the likelihood of clinically relevant subsidence was evaluated using the odds ratio (OR) from univariate logistic regression. Correlations between variables were evaluated using Spearman's rank correlation. All analyses were performed in Python 3.10 (Python Software Foundation) with a significance level of 0.05.

## Results

A total of 82 patients were included in the sample, for a total of 164 hips analyzed, with characteristics shown in Table 1 . There is no evidence of a change in BMI between the 1 ^st^ and 2 ^nd^ surgeries ( *p* = 0.459).

**Table 1:** Sample characteristics

Sex	
Male	43 (52%)
Female	39 (48%)
**Age (years)**	54 ± 10
**Time between surgeries (months)**	32 (25–58)
**BMI**	
1 ^st^ surgery	29 (26–33)
2 ^nd^ surgery	28 (25–32)

**Abbreviation:** BMI: Body Mass Index.

In the first surgery, 21 patients received type I prostheses, and the remaining 61 patients received type II prostheses. A subsidence of 2.36 ± 1.45 mm was observed at the end of 6 months after the first surgery and 1.79 ± 1.27 mm after the second surgery, indicating greater subsidence after the first surgery ( *p* = 0.006). Clinically relevant subsidence was observed in 28 (34.1%) stems in the first surgery and in 13 (15.8%) in the second, representing a 2.75-fold higher incidence in the first surgery ( *p* = 0.008).

The subsidence observed at the end of 6 months was 1.93 ± 1.16 mm in type I prostheses and 2.23 ± 1.57 mm in type II, providing no evidence of greater subsidence in either type ( *p* = 0.181). However, clinically relevant subsidence was observed in 15 (18.2%) type-I stems and 26 (31.7%) type-II, indicating that the chances of clinically relevant subsidence are 2.07 times higher in type-II prostheses ( *p* = 0.050).

The radiographic indices are shown in Table 2 . No evidence of a correlation between subsidence and CEI, CFR, age, or BMI was observed in any stem type. There were also no correlations between these variables and the subsidence observed separately in the first and second surgeries.

**Table 2:** Radiographic characteristics of the hips

	Type I	Type II
**CEI**	35 (2.9–3.8)	3.3 (3.1–3.9)
**CFR (A)**	0.77 (0.69–0.82)	0.77 (0.70–0.85)
**CFR (B)**	0.79 (0.71–0.85)	0.81 (0.69–0.86)
**CFR (C)**	0.63 (0.56–0.74)	0.76 (0.63–0.82)

**Abbreviations:** CEI, cortical enlargement index; CFR, canal fill rate.

## Discussion

The main finding of the study, a pioneer in evaluating femoral stem subsidence in the Brazilian population, is that type-II prostheses are more likely to undergo clinically relevant subsidence. Interestingly, greater subsidence was observed during the first surgery in individuals undergoing bilateral THA. No evidence of a correlation between subsidence and demographic characteristics or radiographic assessments was found.

The risk of subsidence in type-II prostheses observed here is consistent with findings from other studies. In a study conducted at Taiwan's Veterans General Hospital, the incidence of clinically relevant subsidence was 4.8% in type-II prostheses and 2.0% in type-I prostheses. 13

At the Kyushu University Hospital, Japan, there was a mean subsidence of type-I stems of 0.24 mm, against 0.65 mm in type-II stems. In this period, clinically relevant subsidence was observed in 10% of type-II stems and only 1% of type-I stems. 12 In both studies, clinically relevant subsidence rates appear to be lower than those observed in the present study, possibly due to differences between institutional contexts. Tertiary and reference institutions, such as the one in this study, tend to receive cases of greater complexity.

In this sense, the findings presented here reinforce the possible biomechanical advantage of type I stems, even in more challenging populations. This advantage is also supported by data from the US's Total Joint Replacement Registry, which reported a revision rate of 0.5% at 10 years of follow-up for type-I stems, compared with 1.5% for type-II stems, in a database with more than 40 thousand prostheses. 14

In contrast to the previous findings, a retrospective analysis of more than 5 thousand THAs recorded in an Italian regional database found no significant differences in subsidence between type-I and -II stems. 15 Such divergence could be used to illustrate the so-called reproducibility crisis in the medical sciences, 16 but it is fundamental to consider the hypothesis that the magnitude of the effect studied, the relationship between the fixation principle and stem subsidence in this case, varies according to the clinical and population context. In view of this precaution, technical decisions, public policies, and clinical guidelines should be based on data generated in the environment where they will be applied.

In this sense, the analyses presented here support the decision-making of surgeons and managers of the Brazilian national health system. Additionally, this work shows that methodological strategies that increase the sensitivity of the analyses, such as the intraindividual pairing used in this study, are essential to detect subtle effects, such as the benefit observed here in type-I stems, which could go unnoticed in other designs.

The analysis revealed an unexpected finding: individuals who underwent bilateral THA at different times tend to exhibit greater subsidence in the first surgery. Understanding the causes is critical to establish effective strategies to mitigate this effect. We did not observe variations in BMI, which makes it unlikely that BMI is a causal factor. The time between surgeries appears short relative to the age variability observed in the sample, so age does not appear to explain the finding.

Patients undergoing unilateral primary THA show increased physical activity in the years following surgery. 17 However, it is possible that this does not occur after the second surgery in bilateral cases. Thus, future investigations evaluating the level of physical activity and its relationship to stem subsidence in bilateral cases may be enlightening. Considering that the level of intense physical activity appears to increase the risk of revision at 10 years, 18 such an investigation may also help elucidate the causes of failure in unilateral cases.

In this study, no evidence was found that bone quality, stem fit, or demographic characteristics are associated with stem subsidence in any of the types evaluated. Although some investigations have found weak correlations of BMI and age, 19 or only BMI, 8 with stem subsidence, several others have not found any significant correlation. 12 13 18 20 21

Collectively, these findings suggest that although patient characteristics may influence subsidence to some degree, the effect is small and hardly detectable. It is possible that this difficulty is related to the error of the subsidence measurement obtained by radiography. This error is evidenced by negative subsidence reports and cases in which the stem appears to move away from the femur, as observed in all the studies mentioned above, including the present one. Given this low statistical power, it is not surprising that the studies that found correlations have evaluated at least 300 prostheses. The findings of the present study reinforce the conclusion that even in contexts where the effects of proximal femoral radiographic characteristics and patient demographics are not detectable, the principle of stem fixation remains an important mechanical determinant of subsidence.

This work makes an unprecedented contribution to the investigation of the relationship between femoral stem fixation principles and subsidence in a reference service within the highly complex orthopedic treatment of the Brazilian national health system. Additionally, the study identified greater subsidence in the first surgery in bilateral cases, an unprecedented finding.

However, the present study has important limitations. The main one concerns the use of two-dimensional radiographs to measure the subsidence, a technique that is subject to positioning errors and anatomical overlap. Despite the methodological advantage of including patients undergoing bilateral THA, the design was not balanced. The distribution of stem types and the order of surgery were not controlled, making it difficult to distinguish the effects of these two factors.

It should be noted that such balancing, although methodologically desirable, would imply selecting the stem type based on investigative interest rather than clinical criteria, which would be ethically unacceptable. It is important to consider that the Corail stems used, although consistent with the principle of fixation characteristic of type-I stems, present relevant geometric differences in relation to other stems in this category. Thus, the results of the current study may not be fully generalizable to all type-I prostheses.

## Conclusion

Type-II stems have higher risks of clinically relevant subsidence in the first 6 months after primary THA surgery. In bilateral arthroplasties, the subsidence is greater in the first surgery. No correlations were found between stem subsidence and radiographic characteristics of the proximal femur or patient demographic characteristics, regardless of the stem fixation principle.
